# A pilot multiplex salivary transcriptomic analysis to understand the sex-specific effects of maternal opioid use in offspring

**DOI:** 10.1038/s41598-026-49873-6

**Published:** 2026-04-24

**Authors:** Tomoko Kaneko-Tarui, Francesca Carasi-Schwartz, Kiran Singh, Kelsea R. Gildawie, Fair M. Vassoler, Elizabeth M. Byrnes, Elizabeth Yen

**Affiliations:** 1 Woman, Mother + Baby (WoMB) Research Institute, Tufts Medical Center, 15 Kneeland Street, 9th Floor, 02111 Boston, MA USA; 2 Tufts University School of Medicine, Boston, MA USA; 3 Department of Psychology, Simmons University, Boston, MA USA; 4 Department of Comparative Pathobiology, Tufts University Cummings School of Veterinary Medicine, Grafton, MA USA; 5 Department of Pediatrics, Tufts Medicine-Boston Children’s, Boston, MA USA

**Keywords:** Maternal, Offspring, Opioid exposure, Sex differences, Saliva, Multiplex transcriptomics, Diseases, Medical research, Neuroscience, Physiology

## Abstract

Opioid use disorder affects males and females differently, yet the molecular mechanisms in neonates remain understudied. Our laboratory has demonstrated differential sex effects of opioids on select reward and inflammatory pathways related to neonatal feeding behavior that may affect growth and cardiometabolic outcomes. In this pilot observational study, we explored whether gene expression in the reward, energy homeostasis, inflammation, oxidative stress, and neuropathology pathways differed by prenatal opioid exposure and infant sex. Saliva from nine opioid-exposed and nine non-exposed neonates collected within 48 h after birth underwent a multiplex, high-throughput analysis of 72 genes using NanoString’s nCounter^®^ system (NanoString Technologies, Seattle, WA, USA). Despite low RNA abundance in neonatal saliva, experimental conditions were optimized after several trials. Multiplex analysis showed sex-specific expression patterns related to maternal opioid use: male sex was associated with higher expression of genes annotated to reward, inflammation, oxidative stress, feeding, and energy homeostasis pathways, whereas female sex was associated with lower expression of inflammation- and cardiovascular-related transcripts. Future work in larger, independent cohorts will be needed to validate these sex-specific expression patterns and determine their relationships with short- and long-term health outcomes.

**Supplementary Information:**

The online version contains supplementary material available at 10.1038/s41598-026-49873-6.

## Introduction

The incidence of opioid use disorder (OUD) among pregnant individuals has steadily increased^1,2^, accompanied by a parallel surge in neonatal opioid withdrawal syndrome (NOWS). NOWS affects approximately 6–20 per 1,000 live births, depending on the geographic region and population^3–5^. Medications for opioid use disorder (MOUD), including methadone and buprenorphine, are the standard of care for pregnant individuals with OUD^6,7^. These opioid agonist therapies reduce illicit opioid use, improve maternal health, and promote prenatal care. However, both drugs cross the placenta and can influence fetal neurodevelopment, potentially affecting neonatal feeding behavior, immune function, and long-term health outcomes^8–10^. In addition to the clinical management of acute withdrawal in the period after birth, prenatal opioid exposure is also associated with more chronic and long-term effects, including low birth weight, small-for-gestational-age (SGA) status, impaired postnatal growth, and neurodevelopmental delays^9,11–13^. Therefore, maternal OUD is a significant public health concern, with profound implications for neonatal health and developmental outcomes.

Despite the well-documented clinical manifestations and risks, the molecular mechanisms underlying neonatal responses to opioid exposure *in utero* remain understudied. Emerging evidence from both human and animal studies suggests that sex-specific biological responses may play a critical role in shaping the trajectory of these outcomes. For instance, male neonates exposed to opioids *in utero* are more vulnerable to severe NOWS and exhibit poorer cognitive and behavioral outcomes compared to opioid-exposed females^14,15^. These sex differences are likely driven by variations in immune, metabolic, and neurodevelopmental pathways^16^.

Recent studies have begun to elucidate molecular pathways that may mediate these sex-specific effects of opioid exposure. One such pathway involves the dopamine receptor D2 (DRD2), a critical component of the brain’s reward signaling system^17,18^, which has been implicated in opioid-related reinforcement behaviors. In animal models, opioid exposure has been linked to toll-like receptor 4 (TLR4)-related microglial signaling and proinflammatory cascades, alongside changes in reward-related behaviors^19,20^; whether and how these processes translate to humans remains uncertain. Our laboratory was the first to demonstrate sex-specific effects of maternal opioid use on *DRD2* expression as a potential mechanism for aberrant feeding behavior in neonates, particularly males^21^. We also previously observed that higher expression of proinflammatory genes - interleukin-6 (*IL6*), interleukin-1 beta (*IL1β*), and tumor necrosis factor alpha (*TNFα*) occurred alongside markers of white matter injury in opioid-exposed offspring, with greater effects observed in females than in males^22^. Together, these findings highlight that reward signaling and immune activation may be differentially regulated by opioids depending on neonatal sex.

Building on these data, the current pilot study aimed to identify additional candidate genes that may address the critical knowledge gap of understanding the sex-specific effects of maternal opioid use. Leveraging multiplex, high-throughput commercial techniques that have not been previously applied to neonatal saliva, we profiled gene pathways related to opioid use, including inflammation, reward, feeding regulation, oxidative stress, and energy metabolism^16–19,21–23^. In addition to opioid exposure and sex, we also explored whether molecular signatures vary by the types of maternal opioid use (methadone, buprenorphine).

## Results

### Participant characteristics

Following an initial quality check (QC) against the standard for multiplex transcriptomics and further optimization of the transcriptomic experiments (see Methods), samples from 18 neonates (nine opioid-exposed, nine non-exposed) passed QC and were included in the final analysis. As shown in Table 1, SGA occurred only in the opioid-exposed group (33.3% vs. 0%; Fisher’s exact *p* = 0.21), while maternal hepatitis C was more frequent in the opioid-exposed pregnancies (55.6% vs. 0%; *p* = 0.03). Opioid-exposed neonates were also smaller at birth in weight, length, head circumference, and percentiles. Two opioid-exposed neonates (22.2%) developed severe NOWS requiring pharmacotherapy (morphine). The non-exposed group has a higher proportion of females (77.8%) versus the opioid-exposed group (33.3%). The cohort included 10 females (7 non-exposed, 3 opioid-exposed) and 8 males (2 non-exposed, 6 opioid-exposed). Because of the imbalance across sex-by-exposure strata, we provide sex-stratified descriptive clinical characteristics and maternal substance exposure patterns in Supplementary Table S2.

**Table 1:** Maternal and neonatal characteristics by opioid exposure status.

**Characteristics**	**Non-exposed (N=9)**	**Opioid-exposed (N=9)**	**Effect size (95% CI)**	*P* value^1^
**Maternal**				
Age (years)	32.3 (6.1)	32.9 (3.8)	MD 0.54 (−4.69–5.77)	0.83
Gravida	4 (2, 4)	6 (5, 9)	MD 2.05 (0.00–7.50)	**0.02**
Para	1 (1, 5)	2 (2, 3)	MD 1.50 (−3.00–2.00)	0.24
White, n (%)	7 (77.8)	8 (88.9)	OR 2.29 (0.17–30.96)	1.00
Non-Hispanic, n (%)	6 (66.7)	8 (88.9)	OR 4.00 (0.33–48.66)	0.58
Cigarette smoking, n (%)	1 (11.1)	4 (44.4)	OR 6.40 (0.55–74.89)	0.29
Hepatitis C, n (%)	0 (0.0)	5 (55.6)	OR 23.22 (1.04–517.96.04.96)	**0.03**
GBS, n (%)	0 (0.0)	2 (22.2)	OR 6.33 (0.26–152.87)	0.47
Preeclampsia, n (%)	0 (0.0%)	2 (22.2)	OR 6.33 (0.26–152.87)	0.47
GDM, n (%)	0 (0.0%)	0 (0.0%)	NA	1.00
Buprenorphine, n (%)	NA	7 (77.8)	NA	NA
Polysubstance, n (%)	NA	6 (66.7)	NA	NA
**Neonatal**				
Female, n (%)	7 (77.8)	3 (33.3)	OR 0.14 (0.02–1.16)	0.15
Male, n (%)	2 (22.2)	6 (66.7)	OR 7.00 (0.86–50.00.86.00)	0.15
GA (weeks)	37.0 (1.6)	37.6 (1.6)	MD 0.60 (−1.00–2.20)	0.44
Cesarean section	6 (66.7)	4 (44.4)	OR 0.40 (0.06–2.70)	0.64
1-minute Apgar	8.0 (8.0, 9.0)	8.0 (8.0, 8.0)		0.84
5-minute Apgar	9.0 (9.0, 9.0)	9.0 (8.0, 9.0)		0.56
BW (g)	2955.1 (433.8)	2721.4 (624.9)	MD −233.70 (−776.64–309.24)	0.37
BW percentile	62.9 (49.0, 66.0)	28.0 (4.0, 46.0)		**0.02**
Length (cm)	48.3 (2.1)	46.8 (4.5)	MD −1.50 (−5.13–2.13)	0.38
Length percentile	55.0 (37.0, 73.0)	23.0 (10.0, 37.0)		0.07
HC (cm)	33.7 (0.9)	33.0 (2.8)	MD −0.70 (−2.90–1.50)	0.49
HC percentile	74.0 (60.0, 75.0)	21.0 (12.3, 49.0)		0.06
SGA, n (%)	0 (0.0)	3 (33.3)	OR 10.23 (0.45–233.25)	0.21
CPAP at birth, n (%)	5 (55.6)	5 (55.6)	OR 1.00 (0.16–6.42)	1.00
Respiratory issues*, n (%)	2 (22.2)	3 (33.3)	OR 1.75 (0.22–14.22)	1.00
NICU admission**, n (%)	2 (22.2)	5 (55.6)	OR 5.83 (0.70–48.87)	0.15
Antibiotics 48-hours, n (%)	2 (22.2)	0 (0.0)	OR 0.04 (< 0.01–2.93)	0.33
Pneumonia, n (%)	0 (0.0)	0 (0.0)	NA	1.00
Sepsis, n (%)	0 (0.0)	0 (0.0)	NA	1.00
Pharmacotherapy (morphine), n (%)	NA	2 (22.2)	NA	NA

^1^Welch two sample t-test (normally distributed continuous measures); Wilcoxon rank sum test (non-normally distributed continuous measures); Fisher’s exact test (categorical measures with expected cell counts < 5). GBS: group B streptococcus colonization, GDM: gestational diabetes mellitus, GA: gestational age; BW: birth weight; HC: head circumference, SGA: small for gestational age; NICU: neonatal intensive care unit, CPAP: continuous positive airway pressure. Data are presented as mean (standard deviation) for normally distributed continuous measures, median (interquartile ranges) for non-normally distributed continuous measures, or N (%) for categorical measures. Effect size is reported as MD (mean difference) or OR (odds ratio). Bolded p values indicate nominal *p* < 0.05 (exploratory). ORs are not estimable when both groups have zero events. NA indicates either not estimable (both groups have zero events) or not applicable (characteristics defined only among opioid-exposed participants).

*Respiratory issues: transient tachypnea in the newborn (TTN), respiratory distress syndrome (RDS), meconium aspiration syndrome (MAS); **NICU admission is related to the respiratory issues (2 non-exposed and 3 opioid-exposed) and pharmacotherapy need (2 opioid-exposed).

### Global transcript patterns in neonatal saliva across exposure and sex strata

We quantified normalized NanoString gene expression in neonatal saliva samples (*n* = 18) from opioid-exposed (Exp) and non-exposed (Non-Exp) groups, further stratified by sex (F: females, M: males; Non-Exp_F *n* = 7; Non-Exp_M *n* = 2; Exp_F *n* = 3; Exp_M *n* = 6). For reference, the underlying sample-level raw count matrix is provided in Supplementary Table S1. Unsupervised hierarchical clustering of row z-scored expression values across all samples demonstrated substantial inter-individual heterogeneity and revealed coordinated expression modules spanning inflammatory/innate immune mediators (e.g., chemokine/cytokine-related transcripts), *MAPK/NF-κB* signaling components, and metabolic signaling genes (Supplementary Figure S1). This cohort-wide heatmap provides an inclusive overview of sample-level variability after background correction and housekeeping normalization.

**Fig. 1:**
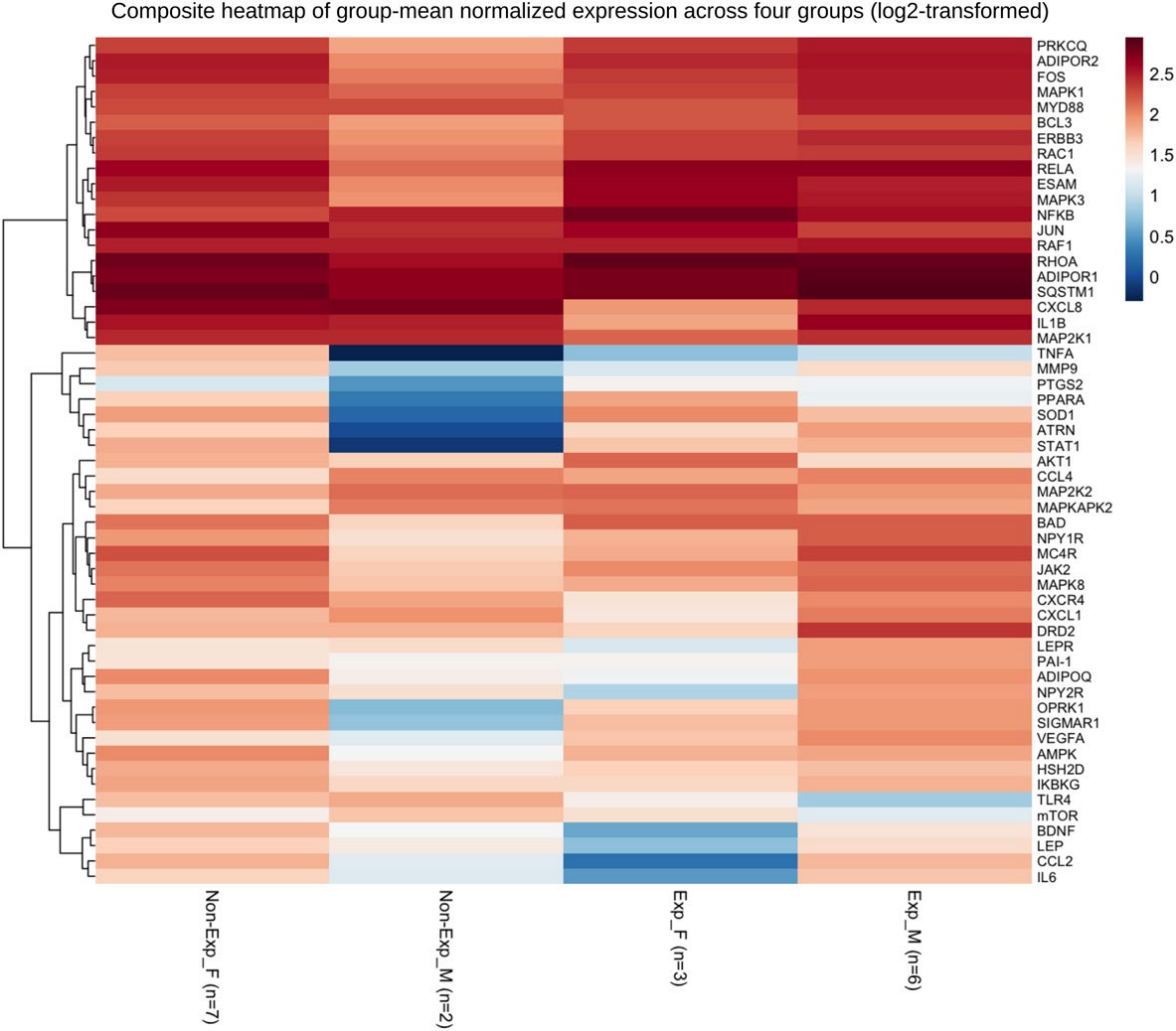
Heatmap of differentially expressed transcripts across exposure strata. Composite heatmap showing group-mean normalized expression. ROSALIND-generated log2-transformed normalized expression values were averaged within each of four groups (Non-Exp_F, Non-Exp_M, Exp_F, Exp_M). Columns represent group means (sample size per group indicated in the column labels), and rows represent genes. Genes were hierarchically clustered (Euclidean distance, complete linkage), while the column order was fixed by group. For visualization, the four group-mean values for each gene were scaled to row-wise z-scores; colors therefore indicate relative expression (higher vs. lower within each row) rather than absolute expression levels. This figure provides a cohort-level summary that complements the sample-level heatmap in Supplementary Fig. S1.

To summarize cohort-level patterns while reducing the influence of sample-to-sample variability, we next computed group-mean expression profiles within each sex-by-exposure stratum and visualized them as a composite heatmap (row z-score across strata; Fig. 1). This composite view highlights broad shifts in the mean expression landscape across strata, including differential behavior among immune/inflammatory signaling genes versus metabolic signaling genes. Given the modest cohort size and the imbalance in stratum counts (particularly Non-Exp_M), these visual patterns serve as descriptive summaries to motivate targeted, hypothesis-driven comparisons in downstream analyses rather than as standalone evidence of differential expression.

### Gene expression differences by opioid exposure

Comparing opioid-exposed with non-exposed neonates, we observed differential expression in several inflammation- and neurodevelopment-related genes (Table 2). *PTGS2* expression was higher in the exposed group (fold change: 3.00; *p* = 0.02), whereas *CCL2* and *BDNF* expression were lower (fold changes: −3.42 and − 2.38, respectively; *p* < 0.05).

**Table 2:** Differential gene expression by opioid exposure.

By Exposure	Genes	Abbreviation	Pathways/diseases*	Fold Change	*P* value
All (9 exp. vs. 9 non-exp.)	*CCL2*	C-C motif chemokine ligand 2, monocyte chemoattractant protein-1 (*MCP-1*)	Immunoregulatory, inflammation, neurodegenerative disorder, cancer, cardiovascular diseases	−3.42	**0.03**
	*BDNF*	Brain derived neurotrophic factor	Neuronal development, cognition, memory, learning, neuropsychiatry	−2.38	**0.03**
	*PTGS2*	Prostaglandin-endoperoxide synthase 2, cyclooxygenase 2 (*COX 2*)	Inflammation, prostaglandin biosynthesis	3.00	**0.02**
Females (3 exp. vs. 7 non-exp.)	*CCL2*	C-C motif chemokine ligand 2, monocyte chemoattractant protein-1 (*MCP-1*)	Immunoregulatory, inflammation, neurodegenerative disorder, cancer, cardiovascular diseases	−8.14	**0.03**
	*CXCR4*	C-X-C motif chemokine receptor 4	Immune response, neurological functions, cancer progression	−6.21	**0.01**
	*IL6*	Interleukin 6	Inflammation, immune response, cancer progression, obesity, diabetes	−5.29	0.05
Males (6 exp. vs. 2 non-exp.)	*DRD2*	Dopamine receptor 2	Reward sensitivity, locomotion, cognition, emotion regulation	6.04	0.05
	*RELA*	REL proto-oncogene, NF-ĸB subunit, p65	Oxidative stress, inflammation, autoimmune disorders, cancer	5.94	**< 0.01**
	*MC4R*	Melanocortin 4 receptor	Energy homeostasis, body weight and feeding regulation, obesity	5.54	0.05
	*FOS*	Fos proto-oncogene	Immune response, inflammation, cancer	4.96	**0.04**
	*PTGS2*	Prostaglandin-endoperoxide synthase 2	Inflammation, prostaglandin biosynthesis	4.33	0.05
	*SQSTM1*	Sequestosome 1 (p62)	Immunity, neurodegenerative disorders, cancer	3.14	**0.02**
**By Opioid Type**	**Genes**	**Abbreviation**	Pathways/diseases*	**Fold Change**	**P value**
2 Methadone vs. 7 Buprenorphine	*CXCR4*	C-X-C motif chemokine receptor 4	Immune response, neurological functions, cancer progression	9.38	**< 0.01**
	*TNF*	Tumor necrosis factor	Neuronal development, cognition, memory, learning, neuropsychiatry	5.40	**0.04**
	*SQSTM1*	Sequestosome 1 (p62)	Immunity, neurodegenerative disorders, cancer	3.14	**0.02**
**By Tx Requirement**	**Genes**	**Abbreviation**	Pathways/diseases*	**Fold Change**	**P value**
2 Tx vs. 7 No Tx	*MAPK1*	Mitogen-activated protein kinase 1	Cell proliferation and growth factor–responsive transcription, cancer	2.94	**0.01**
	*RELA*	REL proto-oncogene, NF-ĸB subunit, p65	Oxidative stress, inflammation, autoimmune disorders, cancer	2.96	**0.02**
	*RAC1*	Ras-related C3 botulinum toxin substrate 1	Cell migration, invasion, cancer metastasis, oxidative stress, inflammation	2.07	**0.02**
	*ADIPOR1*	Adiponectin receptor 1	Insulin sensitivity, glucose uptake, fatty acid oxidation, energy metabolism, obesity, metabolic syndrome	3.57	**0.02**

*source: Rosalind^®^ Academy version 3.39.13.1 (https://rosalind.bio/academy); Exp: opioid-exposed, Tx: pharmacotherapy.

Pathway/disease annotations were obtained from ROSALIND^®^ Academy (ontology-based curated labels). In this pilot study, these annotations are provided for descriptive context only and do not imply clinical disease phenotypes, diagnoses, or disease risk in the study cohort.

When we stratified the analyses by sex, we observed sex-specific expression patterns in relation to maternal opioid use. Within the female cohort, maternal opioid use was associated with lower expression of inflammation-related genes, including *CCL2*, *CXCR4*, and *IL6* (fold changes: − 8.14 to − 5.29; *p* = 0.01–0.05). Within the male cohort, maternal opioid use was linked to higher expression of *DRD2*, *RELA*, *MC4R*,* FOS*,* PTGS2*, and *SQSTM1* (fold changes: 3.14 to 6.04; *p* < 0.01–0.05). These genes map to pathways related to reward signaling, oxidative stress, inflammatory signaling, and energy homeostasis. We note that pathway annotation resources may return broad disease-category labels for some genes. In this pilot study, these labels are reported as ontology-based annotations only and are not interpreted as evidence of clinical disease phenotypes or disease risk in the cohort.

### Differences by types of opioid exposure

In an exploratory subgroup comparison among opioid-exposed neonates, we observed higher expression of immune-related genes in methadone-exposed neonates (*n* = 2) compared with buprenorphine-exposed neonates (*n* = 7). Specifically, *CXCR4* and *TNF* showed higher expression in the methadone group (fold changes: 9.38 and 5.40; *p* < 0.05), as did *SQSTM1*, a key mediator of autophagy and cellular stress responses (fold change: 3.14, *p* = 0.02) (Table 2). Given the small sample size, these findings should be interpreted cautiously and considered hypothesis-generating. Some enriched pathway terms fall under cancer-related ontology categories; these labels reflect database annotations and should not be interpreted as evidence of malignancy-related biology in this cohort.

### Differences by pharmacotherapy requirement

Among opioid-exposed neonates (*n* = 9), two developed severe NOWS requiring pharmacotherapy (morphine), and seven did not require pharmacotherapy. Exploratory within-exposed comparisons identified higher expression of inflammatory, oxidative stress signaling, metabolic-related, and insulin sensitivity genes in the pharmacotherapy group, including *MAPK1* (fold change 2.94, *p* = 0.01), *RELA* (fold change 2.96, *p* = 0.02), *RAC1* (fold change 2.07, *p* = 0.02), and *ADIPOR1* (fold change 3.57, *p* = 0.02) (Table 2).

### Sex-specific differences in gene expression

Independent of exposure status, sex-stratified analysis across all neonates showed that males had lower expression of metabolic genes such as *PPARα*, *AKT1*, and *AMPK* compared to females (fold changes: − 4.10 to − 2.46; all *p* < 0.05), and greater expression of prostaglandin biosynthesis factor *PTGS2*, angiogenic and atherosclerotic factor *VEGFα*, and metabolic homeostasis *LEPR* (fold changes: 2.5 to 3.75, *p* < 0.05).

Among non-exposed neonates, *RELA* was downregulated in males compared to females (fold change: − 4.30, *p* = 0.02), suggesting a lower baseline inflammatory state. However, among opioid-exposed neonates, males showed greater expression of inflammatory and immune pathway genes, including *PTGS2*, *TLR4*, *IL1β*, *CXCR4*, and *CXCL8* (fold changes: 2.20 to 6.50; all *p* < 0.05), in line with sex-specific immune and neuroinflammatory transcriptional signatures within the exposed group (Table 3).

**Table 3:** Differential gene expression by sex.

By Sex	Genes	Abbreviation	Pathways/diseases*	Fold Change	*P* value
All (8 males vs. 10 females)	*PPARA*	Peroxisome proliferator activated receptor alpha	Energy, cholesterol, and lipid metabolism, glucose homeostasis, immune, inflammation, obesity, atherosclerosis	−4.10	**0.01**
	*AKT1*	AKT serine/threonine kinase 1	Cell metabolism, insulin signaling, cancer,	−3.24	**0.01**
	*AMPK*	AMP-activated protein kinase	Energy metabolism, fatty acid oxidation, inflammation, diabetes, obesity, metabolic disorder	−2.46	**0.04**
	*PTGS2*	Prostaglandin-endoperoxide synthase 2	Inflammation, prostaglandin biosynthesis	3.75	**< 0.01**
	*VEGFA*	Vascular endothelial growth factor A	Angiogenesis, atherosclerosis, hypoxia-induced neovascularization, obesity, tumor growth, cancer	3.15	**0.03**
	*LEPR*	Leptin receptor	Energy homeostasis, metabolism, insulin signaling, glucose homeostasis, obesity, cancer, metabolic syndrome, cardiovascular disease	2.5	**0.03**
Non-exp. (2 males vs. 7 females)	*RELA*	REL proto-oncogene, NFĸB subunit, p65	Oxidative stress, inflammation, autoimmune disorders, cancer	−4.30	**0.02**
Exp. (6 males vs. 3 females)	*PTGS2*	Prostaglandin-endoperoxide synthase 2, cyclooxygenase 2 (*COX 2*)	Inflammation, prostaglandin biosynthesis	6.50	**0.01**
	*TLR4*	Toll-like receptor 4	Innate immunity, pathogen recognition, inflammation, microglia activation, neuroinflammation, cancer, autoimmune disorders, neurodegeneration	5.88	0.05
	*IL1B*	Interleukin 1 beta	Inflammation, neuroinflammation, cancer, diabetes, bipolar disorder, Alzheimer’s disease	5.49	**0.04**
	*CXCR4*	C-X-C motif chemokine receptor 4	Immune response, neurological functions, cancer progression	2.29	**0.03**
	*CXCL8*	C-X-C motif ligand 8	Inflammation, cancer, neurodegeneration, neuroinflammation, Parkinson’s disease	2.20	0.05

*source: Rosalind^®^ Academy version 3.39.13.1 (https://rosalind.bio/academy); Exp: opioid-exposed.

Disease terms (e.g., neurodegenerative disorder) are database-derived annotation labels used for gene-set categorization only and do not imply clinical diagnosis or disease risk in this cohort.

## Discussion

In this study, we observed exploratory, sex-specific differences in salivary gene expression among neonates prenatally exposed to maternal methadone and buprenorphine. Our findings are hypothesis-generating and require further validation in larger cohorts. However, our study is the first to leverage a high-throughput, commercial multiomic profiling platform for neonatal saliva using the nCounter^®^ Analysis System (NanoString Technologies, Seattle, WA, USA). By adapting this platform to saliva, we observed distinct expression patterns across inflammation, reward signaling, and energy regulation pathways, generating hypotheses about pathways associated with sex-specific differences in neonatal outcomes.

Our findings are consistent with prior observations from animal models and emerging human data, while introducing neonatal saliva as a feasible biospecimen for molecular analysis. Given the pilot design and limited statistical power, between-group differences are interpreted as exploratory. Miller et al. reported reduced neutrophils and inflammatory cytokines in the umbilical cord blood of opioid-exposed neonates compared to those in non-exposed neonates^24^. In contrast, Newville et al. observed elevated proinflammatory cytokines and chemokines, along with heightened immune reactivity, in methadone-exposed rats^25^. Taken together, our results add to prior work by describing sex-specific patterns of inflammatory gene expression in relation to maternal opioid use. Specifically, the patterns of lower *CCL2* and *BDNF* expression, alongside greater *PTGS2* expression, suggest a heterogeneous inflammatory signature that may reflect a mixture of biological and environmental influences. For instance, the cohort-level reduction in *CCL2* is likely due to the lower expression in females. Conversely, the cohort-level increase in *PTGS2* aligns with the increase we observed in males. These findings suggest that sex may modify the relationship between maternal opioid use and inflammatory gene expression, as reflected in the sex-specific molecular patterns observed here. These alterations span pathways related to inflammation, immune regulation, cognition, metabolic homeostasis, and long-term neurodevelopmental outcomes.

Therefore, our pilot study highlights the need to include sex as a biological variable in understanding the impact of maternal opioid use on offspring. The greater expression of *DRD2* in opioid-exposed males in the current study aligns with our published data showing the male-predominant effects of opioids on heightened reward signaling and feeding behavior^19^. Of interest is the greater expression of *MC4R* in opioid-exposed males. *MC4R* is critical to feeding and metabolism and is often higher in a satiated state^26^. In our data, the concurrent increase in *DRD2* and *MC4R* expression in opioid-exposed neonates fits with altered reward- and energy homeostasis-related transcription^27^. Whether these patterns track with feeding phenotypes will require direct assessment in a larger cohort. Higher *RELA* and *MC4R* expression in exposed males suggests involvement of oxidative stress and energy homeostasis pathways; however, our observational designs do not allow causal inference. Although these results require validation in larger samples, they provide biological plausibility and a rationale for future studies to directly test the link between sex-specific molecular patterns and clinical phenotypes. If validated and proven, this differential gene expression can help identify effective interventions and personalized care.

Stratification by sex (Table 3) provides further evidence of the distinct molecular response to *in utero* opioid exposure between male and female neonates. Opioid-exposed males exhibited upregulation of inflammation and neuroinflammation, as evidenced by increased expression of *TLR4*,* PTGS2*,* IL1β*,* CXCR4*, and *CXCL8*. While opioids act via opioid receptors, animal data have demonstrated the non-neuronal effects of opioids by binding with TLR4 in microglia and the release of chemokines and cytokines, and subsequent reinforcement of reward signaling^18^. We observed higher expression of both *TLR4* and *DRD2* in opioid-exposed males. This co-occurrence is consistent with potential involvement of neuroimmune pathways and supports follow-up in mechanistic and longitudinal studies.

In contrast, opioid-exposed females showed marked downregulation of genes in the inflammation and immune pathways. Our findings align with previous evidence that female neonates may possess intrinsic immunological advantages and resilience to prenatal stressors^28^ and that male sex confers a greater vulnerability to adverse prenatal and perinatal effects^29^. These sex-specific signatures may be relevant to variability in NOWS severity and later neurodevelopment, but larger, longitudinal studies will be needed to evaluate these associations.

Another important observation, albeit limited by our small sample size, is the differential gene expression based on the type of maternal opioid use. Neonates exposed to methadone demonstrated upregulation of genes in the inflammatory and immune pathways compared to those exposed to buprenorphine. Based on studies showing worse clinical outcomes in neonates exposed to methadone relative to buprenorphine (e.g., more severe NOWS and more extended hospital stays)^30–32^, the molecular data presented here may suggest a biological basis for these clinical trends. In an exploratory analysis restricted to opioid-exposed neonates, those who required pharmacologic treatment for NOWS showed increased expression of *MAPK1* and *RELA*, along with *RAC1* and *ADIPOR1.* These genes converge on pathways implicated in cellular stress responses and inflammatory signaling (e.g., *MAPK* and *NF-kB*–related signaling) as well as metabolic regulation (adiponectin receptor signaling). Although underpowered, these preliminary signals are biologically plausible given that severe NOWS reflects greater physiologic (i.e., feeding) dysregulation in the early postnatal period. Importantly, because the requirement for pharmacotherapy may also correlate with exposure intensity, polysubstance use, and sex distribution within this small pilot cohort, future studies with larger samples and richer covariate structures are needed to determine whether these transcriptional patterns independently predict NOWS severity/pharmacotherapy requirement.

This study is the first to use neonatal saliva on a high-throughput, commercial transcriptomic platform. Despite the initial assay challenges, our team successfully optimized the experimental conditions to generate the current data. The technical descriptions reported in this study serve as proof of concept that using microquantities of neonatal saliva for multiplexed gene analyses is feasible and even desirable. Neonatal research is often limited procedures available to generate data; therefore, the field must leverage the least invasive method possible to ensure safe and robust research in this population.

In addition to the technical novelty, our molecular findings provide intriguing findings that can be utilized in future studies examining the mechanistic and clinical relevance of maternal opioid use in offspring. First, our observations are in line with prior work implicating TLR4-related signaling in opioid-associated neuroimmune interactions^19,33^, although mechanistic interpretation is beyond the scope of this study. Second, whether early differential expression of *DRD2* predisposes to aberrant reward signaling and risk of substance use in adulthood is unknown; we frame this as a hypothesis for future longitudinal studies. Third, the enrichment of metabolism-related pathways among opioid-exposed neonates—especially males—warrants follow-up to determine whether early expression pattens correlate with birth weight, feeding behavior, growth trajectories, or cardiometabolic outcomes. Fourth, while the significance of cancer involvement in the pathways affected by maternal opioid use is unclear, future longitudinal studies should examine the risk of malignancy related to *in utero* and childhood opioid exposure. This is especially important given the higher risk of cancer and cancer-related mortality in people with chronic opioid use^34–36^, although a meta-analysis cautioned against the risk of bias in overestimating the overall effect of opioid use on cancer outcomes^36^. Finally, our pilot study using drops of neonatal saliva highlights the importance of including sex as a biological variable. The current data align with reports of sex differences in risks and outcomes related to OUD, although our study was not designed to establish causal impacts on offspring outcomes. A clear understanding of sex differences may help inform future efforts towards more individualized care for neonates with NOWS, but translation will require replication and outcome-linked studies. From a translational perspective, our data indicate that salivary transcripts could be explored as candidate biomarkers for risk stratification in opioid-exposed neonates. If validated, such markers could potentially inform monitoring or follow-up strategies (e.g., neurodevelopmental follow-up), but this will require prospective evaluation.

Limitations of the current study include the small sample size, which limits statistical power and increases susceptibility to confounding. Although we performed an exploratory differential gene expression comparison by pharmacotherapy requirement within the opioid-exposed group, these subgroup results (2 treated vs. 7 untreated) should be interpreted cautiously and require replication in larger cohorts. The small sample size and single-center design also limit our generalizability. The use of saliva, while non-invasive and clinically practical, captures a subset of systemic molecular activity and may not fully reflect brain-specific processes. Additionally, potential confounding by maternal comorbidities (e.g., hepatitis C, polysubstance use) and unmeasured environmental exposures (e.g., socioeconomic status) cannot be excluded and should be included in future studies with larger sample sizes. Finally, cross-sectional design precludes conclusions about long-term consequences, underscoring the need for longitudinal follow-up. Because some tools output broad disease-category terms, we report them only as annotations and do not infer clinical or mechanistic significance. Our interpretation, therefore, focuses on convergent signals across immune/inflammatory and neurodevelopmental pathways, consistent with prior literature on prenatal opioid exposure.

Future research should expand sample sizes to enable the characterization of gene expression data by important variables such as the need for pharmacotherapy and poly vs. monosubstance exposure. Multimodal platforms, such as additional tissue types (cord blood or placenta), neuroimaging data, and developmental screening tests, will enable a better understanding of the associations with neurodevelopmental outcomes. Given the differential expression on cardiovascular pathways, future studies should also focus on comprehensive cardiometabolic evaluations, including blood pressure and anthropometric measurements. Integrating multi-omics approaches—including epigenetics and proteomics—may further elucidate sex-specific biological programming in opioid-exposed neonates. Importantly, future studies should evaluate whether salivary gene expression profiles can serve as reliable predictors of clinical outcomes or treatment response in neonates with NOWS. Our study also serves as a foundation for a collaboration with preclinical researchers using non-human models to examine the mechanistic underpinnings of maternal opioid use.

In conclusion, this pilot observational study provides proof of concept for the feasibility of using neonatal saliva as a non-invasive approach for exploratory biomarker discovery. Prenatal opioid exposure was linked to sex-specific molecular patterns in neonates, suggesting that biological sex may play an important role in shaping neonatal vulnerability to *in utero* opioid exposure and potentially contributing to the variable severity of NOWS and long-term developmental outcomes. Moreover, differential expression patterns based on the type of opioid—methadone versus buprenorphine—highlight the importance of individualized approaches to maternal treatment. Given the small sample size, our findings should be interpreted cautiously and should be confirmed in larger, adequately powered cohorts.

## Methods

### Study design and participants

This pilot observational study used a prospective cohort design to investigate differences in gene expression between male and female neonates, comparing those with prenatal opioid exposure to those without. Inclusion criteria were neonates born at ≥ 34 weeks’ gestational age (GA) at Tufts Medical Center in Boston, Massachusetts, between February and November 2023. The study cohort included 18 neonates, nine neonates with prenatal opioid exposure (opioid-exposed group) and nine neonates without such exposure (non-exposed group), matched by GA. The opioid-exposed group was defined based on electronic medical record documentation of either maternal OUD on either buprenorphine or methadone throughout the entire pregnancy by verbal or toxicology screening and/or positive infant urine and meconium toxicology testing. Isolated intrapartum opioid analgesia was not classified as prenatal opioid exposure. The non-exposed group consisted of neonates born to mothers with no known opioid use during pregnancy. Exclusion criteria included: major congenital anomalies, genetic/chromosomal abnormalities, clinical chorioamnionitis, or gestational age/GA < 34 weeks.

Maternal demographic and clinical data—including race, ethnicity, delivery type, opioid type, group B streptococcus (GBS) colonization status, hepatitis C status, cigarette smoking, and health comorbidities, such as preeclampsia, gestational diabetes—were abstracted from the electronic medical record using prespecified definitions. Neonatal characteristics and outcomes—including GA, sex, birth weight, length, head circumference and corresponding percentiles, 1- and 5-minute Apgar scores, small-for-gestational-age (SGA) status, resuscitation at birth, NICU admission/diagnoses, antibiotic exposure, and neonatal illnesses, such as respiratory issues, sepsis, pneumonia, and pharmacotherapy requirement—were abstracted using the same approach.

### Ethical approval and consent to participate

The study was approved by the Tufts Medical Center Institutional Review Board. All procedures were performed in accordance with institutional and national research committee ethical standards, the Declaration of Helsinki, and relevant guidelines and regulations. Written informed consent for study participation was obtained from a parent or legal guardian of each neonate prior to enrollment. No identifiable images or personal data are included in this manuscript, and all HIPAA identifiers have been removed.

### Sample collection and RNA extraction

Saliva samples were collected from neonates within 48 h of birth using our established techniques^37^. Briefly, saliva was collected using a 1-milliliter (mL) insulin syringe (Becton, Dickinson and Company, Franklin Lakes, NJ) attached to the low-pressure wall suction for 15–30 s. To minimize breast milk and associated maternal RNA contamination, saliva was collected before feeding or at least 30 min after feeding. Saliva was immediately placed in 250 µl (µL) RNAprotect Saliva Reagent (Qiagen, Hilden, Germany) to minimize RNA degradation. Total RNA was isolated using the RNeasy Micro Kit (Qiagen, Hilden, Germany) according to the manufacturer’s protocols, which were optimized for low-input, saliva-derived specimens. On-column DNase treatment was performed using RNase-free DNase I (Qiagen) to minimize DNA contamination. Once RNA was extracted, the total RNA was stored at −80 °C pending gene expression analysis.

### Gene expression analysis

mRNA expression profiling was performed using the NanoString nCounter Analysis System (NanoString Technologies, Seattle, WA), a direct, multiplexed platform for quantifying gene expression. A custom CodeSet was designed to target 72 genes of interest, including selected housekeeping genes used for normalization. Detailed probe annotation and target gene information are available in the Gene Expression Omnibus (GEO) under accession number GSE327911. Sample-level raw NanoString counts for all targets, including housekeeping genes are provided in Supplementary Table S1.

To prepare samples for hybridization, the NanoString Low RNA Input Kit (NanoString Technologies, Seattle, WA) was used according to the manufacturer’s instructions. We subsequently determined that eight pre-amplification cycles were optimal for amplifying the mRNA transcripts using neonatal saliva, which is known to have a low starting mRNA level^38^. The nCounter assay employs a molecular barcoding system, in which specific probes hybridize directly to target mRNAs. Each probe pair includes a capture probe and a reporter probe containing a unique fluorescent barcode for quantification. Before conducting the main experiments, we performed preliminary optimization tests under varying pre-amplification cycle numbers and hybridization durations. For the final protocol, pre-amplified RNA was hybridized with the Reporter CodeSet and Capture ProbeSet at 65 °C for 22 h, based on preliminary optimization testing.

### Quality control, normalization, and low-abundance targets (ROSALIND)

Raw RCC files were analyzed in ROSALIND^®^ (ROSALIND, Inc., San Diego, CA, USA) using the nCounter Advanced workflow for QC, normalization, and downstream analyses. Across 36 assay instances (RCC files), including technical replicates and re-runs, 18 unique neonatal saliva samples (one per neonate; *n* = 18) passed QC and were included in the final analyses; the remaining 18 QC-flagged assay instances were excluded prior to normalization. Samples were excluded if flagged for standard nCounter QC metrics, including imaging quality (field-of-view registration ≤ 0.75), binding density outside the recommended range for the nCounter SPRINT (0.1–1.8), positive control linearity (R² <0.95), or limit of detection failure (POS_E not exceeding the negative-control background threshold; mean(Negatives) + 2 × SD(Negatives) > POS_E).

Normalization was performed in ROSALIND using the housekeeping genes previously used in our prior studies (*GAPDH*,* HPRT1*, and *YWHAZ*)^39^. A sample-specific scaling factor was derived from the geometric mean of housekeeping-gene counts, and normalized values were then log2-transformed.

Because RCC files provide digital counts for all targets, no imputation was performed; low-abundance targets were retained and interpreted relative to negative-control background/LOD, and samples failing LOD were excluded as above.

### Statistical analysis

This observational study was designed as a pilot analysis. Descriptive statistics are presented as mean (SD) for approximately symmetric continuous variables, median (IQR) for skewed continuous variables, and n (%) for categorical variables. When variables had missing values, denominators reflect available data (n/available). For Table 1 comparisons, categorical variables were compared using two-sided Fisher’s exact tests, and continuous variables summarized as mean (SD) were compared using Welch’s t-tests. When individual-level data were available, and distributions were skewed, Mann–Whitney U tests were used. Effect sizes are reported as odds ratios (OR; opioid-exposed relative to non-exposed) for categorical variables and mean differences (MD; exposed minus non-exposed) for continuous variables, each with 95% confidence intervals (CI). Given the small sample size, inferential results are interpreted cautiously and primarily used to guide future hypothesis-driven studies rather than to support definitive conclusions.

### Heatmap visualization

Normalized NanoString counts (log2-transformed after normalization, as described above) were visualized as heatmaps. For Supplementary Fig. S1, normalized expression values for all samples were displayed with transcripts scaled by row z-scores to emphasize relative expression across samples. For Fig. 1, normalized expression values were first summarized as group means within each sex-by-exposure stratum (non-exposed female, non-exposed male, opioid-exposed female, opioid-exposed male), and transcripts were similarly scaled by row z-scores. Heatmaps are presented as descriptive visual summaries and were not used for statistical inference.

### Sample size and power

No formal a priori power or sample size calculation was performed. This work was conceived as an observational pilot study to assess the feasibility of multiplex, high-throughput analysis using neonatal saliva, NanoString nCounter targeted mRNA quantification, and pre-specified sex-stratified analyses in a clinically rare exposure group. Because reliable estimates of variance and effect size for these targeted transcripts in neonatal saliva were not available at study initiation, the sample size was determined by feasibility and availability of eligible, well-phenotyped specimens. The resulting data provide preliminary estimates of variability and effect sizes to inform power calculations and sample size determination for future, adequately powered confirmatory studies. Data processing (QC, normalization, and handling of low-abundance targets) and downstream differential expression analyses were performed in ROSALIND^®^ using the nCounter Advanced workflow, as described above.

Differential expression analysis was conducted using a fast methods statistical framework described in the *nCounter Advanced Analysis 2.0 User Manual*, which applies a generalized linear model to estimate fold changes and associated p-values. Multiple testing correction was performed using the Benjamini–Hochberg procedure. Given the pilot nature and the targeted panel size, p values in Tables 2 and 3 should be interpreted cautiously. This generalized linear model (GLM)-based approach is particularly well-suited for NanoString data as it accounts for the discrete nature of count data and accommodates technical variation, thereby enhancing the accuracy and reproducibility of differential expression estimates.

GLMs were prespecified to include the following predictors: sex (male/female) and prenatal opioid exposure (unexposed vs. exposed). For full-cohort analyses, exposure was encoded as a three-level factor (unexposed, methadone, buprenorphine) to avoid collinearity between exposure and medication type. Opioid medication type among exposed pregnancies (methadone vs. buprenorphine) and the need for pharmacotherapy for NOWS (yes/no) were recorded as clinically relevant covariates and evaluated in exploratory secondary analyses within the opioid-exposed group; these comparisons were considered hypothesis-generating given the very small subgroup sizes. Given the sample size, higher-order interactions were limited to the sex × exposure term, which was specified a priori.

To assess sex-specific gene expression patterns, data were stratified by sex, and differential expression analysis was performed separately for male and female samples. This approach aimed to identify sex-specific differences in gene expression, particularly those potentially influenced by prenatal opioid exposure.

## Electronic Supplementary Material

Below is the link to the electronic supplementary material.

Supplementary Material 1

## Data Availability

The NanoString nCounter gene expression data generated in this study, including raw RCC files, processed expression matrices, and associated annotation, have been deposited in the NCBI Gene Expression Omnibus (GEO) under accession number GSE327911.
