# Supplementary material for: A pilot multiplex salivary transcriptomic analysis to understand the sex-specific effects of maternal opioid use in offspring
Source: Sci Rep. 2026 Apr 24;16:18969. doi: 10.1038/s41598-026-49873-6 (PMC13276388; doi:10.1038/s41598-026-49873-6)
Supplement: Supplementary file 1 — Supplementary Material 1 [file 41598_2026_49873_MOESM1_ESM.docx]

**Supplementary Information**

This document provides additional information supporting the reproducibility of the NanoString nCounter targeted mRNA expression assay.

**Supplementary Figure S1**

Heatmap displaying ROSALIND-generated log2-transformed, normalized expression values for individual samples across the four groups (Non-Exp_F, Non-Exp_M, Exp_F, Exp_M). Columns represent individual samples (group membership indicated above), and rows represent genes. For visualization, values were scaled to row-wise z-scores across samples; colors therefore indicate relative expression within each transcript (higher vs lower within each row), rather than absolute expression levels. Genes were hierarchically clustered (Euclidean distance, complete linkage).

**Supplementary Figure S1.** Sample-level heatmap of gene expression in the cohort (log2-transformed, gene-wise Z-score)
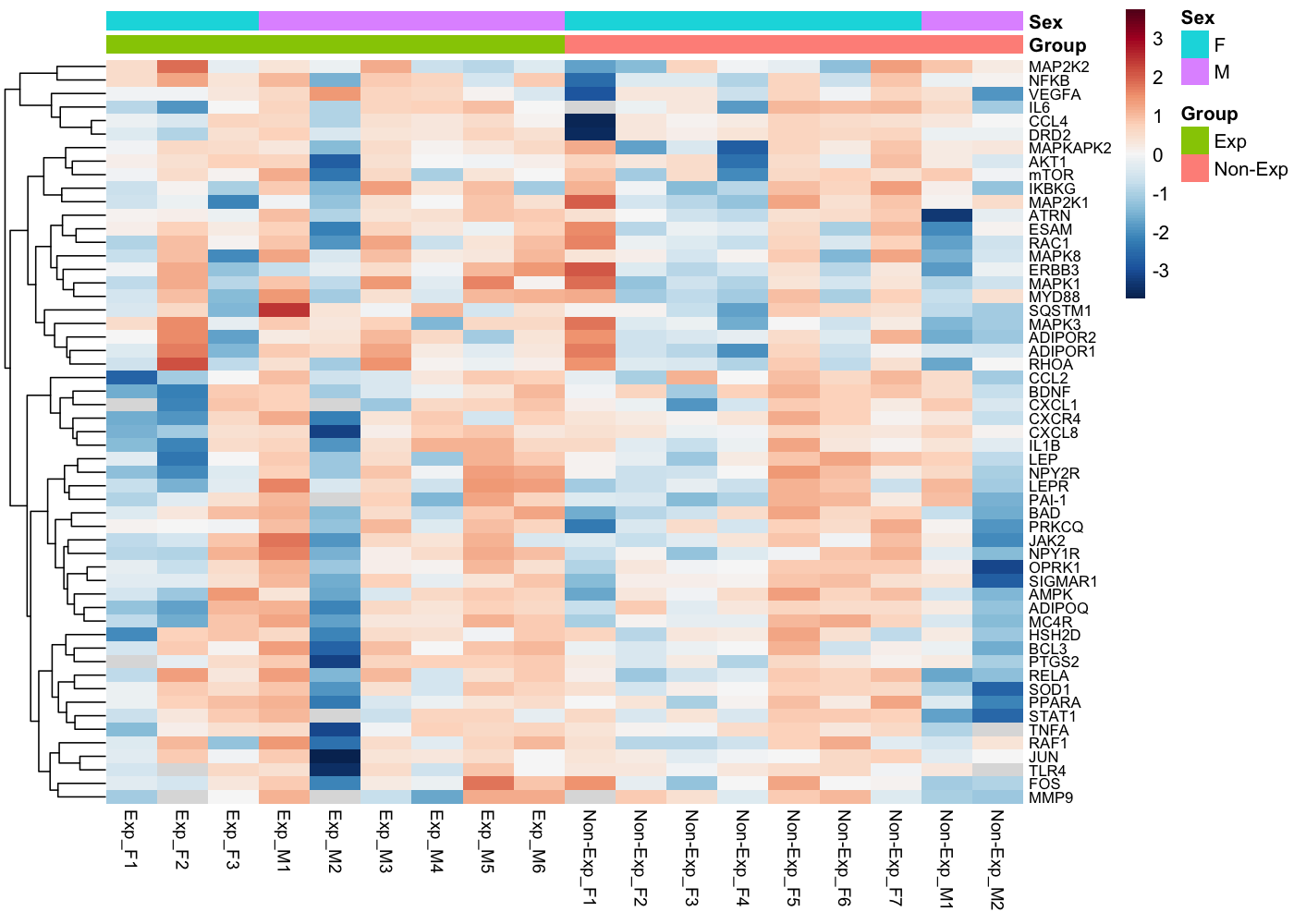


**Supplementary Table S1**

Sample-level raw gene expression values for the multiplex transcriptomic panel.
Raw counts are reported for each target gene in each de-identified sample and are organized by exposure group (Non-Exposed vs Exposed) and sex (Female and Male). Sample identifiers indicate exposure group and sex (NE-F, E-F, NE-M, E-M). Housekeeping targets used for assay normalization/quality control are indicated by an asterisk (*).

**Supplementary Table S2**

Sex-stratified cohort composition and maternal medication/substance exposures.

*Panel A. Sex-by-exposure Distribution.*

| **Exposure status** | **Female (n=10)** | **Male (n=8)** | **P value^1^** |
| --- | --- | --- | --- |
| Non-exposed | 7 (70.0%) | 2 (25.0%) | 0.153 |
| Opioid-exposed | 3 (30.0%) | 6 (75.0%) |  |

*Panel B. Opioid Medication and Substance Exposure by Neonatal Sex.*

| **Opioids/Substance Exposure** | **Female (n=3)** | **Male (n=6)** | **P value^1^** |
| --- | --- | --- | --- |
| Buprenorphine | 3 (100) | 4 (66.7) | 0.50 |
| Methadone | 0 (0.0) | 2 (33.3) | 0.50 |
| Polysubstance | 3 (100.0) | 3 (50.0) | 0.46 |
| Benzodiazepine | 2 (66.7) | 1 (16.7) | 0.23 |
| Amphetamine | 1 (33.3) | 1 (16.7) | 1.00 |
| Cannabis | 1 (33.3) | 1 (16.7) | 1.00 |
| Gabapentin | 1 (33.3) | 0 (0.0) | 0.33 |

^1^Fisher’s exact test (categorical measures with expected cell counts < 5). Data are presented in N (%). P values are exploratory given the pilot sample size.
